# The pro-survival Bcl-2 family member A1 delays spontaneous and FAS ligand-induced apoptosis of activated neutrophils

**DOI:** 10.1038/s41419-020-2676-9

**Published:** 2020-06-18

**Authors:** Robyn L. Schenk, Lahiru Gangoda, Kate E. Lawlor, Lorraine A. O’Reilly, Andreas Strasser, Marco J. Herold

**Affiliations:** 1 The Walter and Eliza Hall Institute of Medical Research, Parkville, VIC Australia; 2 Department of Medical Biology, University of Melbourne, Parkville, VIC Australia; 3 Centre for Innate Immunity and Infectious Diseases, Hudson Institute of Medical Research, Clayton, VIC Australia; 4 Department of Molecular and Translational Science, Monash University, Clayton, VIC Australia; 5 Present Address: Research Institute of Molecular Pathology, Vienna Biocenter, Vienna, Austria

Neutrophils have a short lifespan that is extended after exposure to granulocyte macrophage colony stimulating factor (GM-CSF) or lipopolysaccharide (LPS)^1^. While the survival is regulated by BCL-2 family proteins^2^, it is not known which pro-survival proteins are involved. GM-CSF stimulation in neutrophils upregulates A1, but *A1*-deficient mice showed no defects in this cell type^3^. MCL-1 is critical for the survival of quiescent neutrophils^4,5^, but it is not known whether the same holds true after activation. We hypothesized that A1 and MCL-1 have overlapping roles in the survival of activated neutrophils.

We generated mutant mice deficient for A1 and lacking one allele of *Mcl-1* (*Mcl-1*^*+/–*^*A1*^*–/–*^*). Mcl-1*^*+/–*^*A1*^*–/–*^ mice are grossly normal in the haematopoietic compartment, with only a small reduction in lymphocyte numbers, similar to *Mcl-1*^+/–^ mice^6^ (Supplementary Fig. 1A). Loss of A1 did not cause a survival defect in GM-CSF-stimulated neutrophils. Here, we examined the survival of neutrophils activated with LPS plus GM-CSF from *A1*^*–/–*^, *Mcl-1*^*+/–*^, and *Mcl-1*^*+/–*^*A1*^*–/–*^ mice. Without stimulation, *Mcl-1*^*+/–*^ neutrophils had a significant survival disadvantage compared to their wild-type and *A1*^*–/–*^ counterparts and no further decrease in cell survival was observed in *Mcl-1*^*+/–*^*A1*^*–/–*^ neutrophils (Fig. 1a). Presumably, this increased apoptosis observed in *Mcl-1*^*+/–*^ neutrophils is due to the in vitro conditions, as we saw normal neutrophil numbers in vivo in *Mcl-1*^*+/–*^ or *Mcl-1*^*+/–*^*A1*^*–/–*^ mice (Supplementary Fig. 1B). After activation with LPS plus GM-CSF, the A1^−/−^ and *Mcl-1*^*+/–*^*A1*^*–/–*^ neutrophils exhibited significantly poorer survival, whilst *Mcl-1*^*+/–*^ neutrophils behaved similarly to wild-type cells (Fig. 1b). LPS treatment alone was ineffective at promoting a survival advantage and failed to induce neutrophil blasting or upregulate pro-survival MCL-1 expression (Supplementary Fig. 2A–C). GM-CSF treatment alone promoted survival, blasting, and MCL-1 upregulation in wild-type and *A1*^*–/–*^ cells^3^. GM-CSF is known to induce expression of the TLR4 co-receptor CD14^7^. We observed marked upregulation of CD14 on neutrophils after GM-CSF stimulation, and more so after treatment with GM-CSF plus LPS (Supplementary Fig. 2C). Hence, the survival defect of LPS plus GM-CSF-stimulated *A1*^−/−^ neutrophils could be due to a lack of increased A1 expression, contributing to the survival of activated neutrophils^8,9^.

**Fig. 1:**
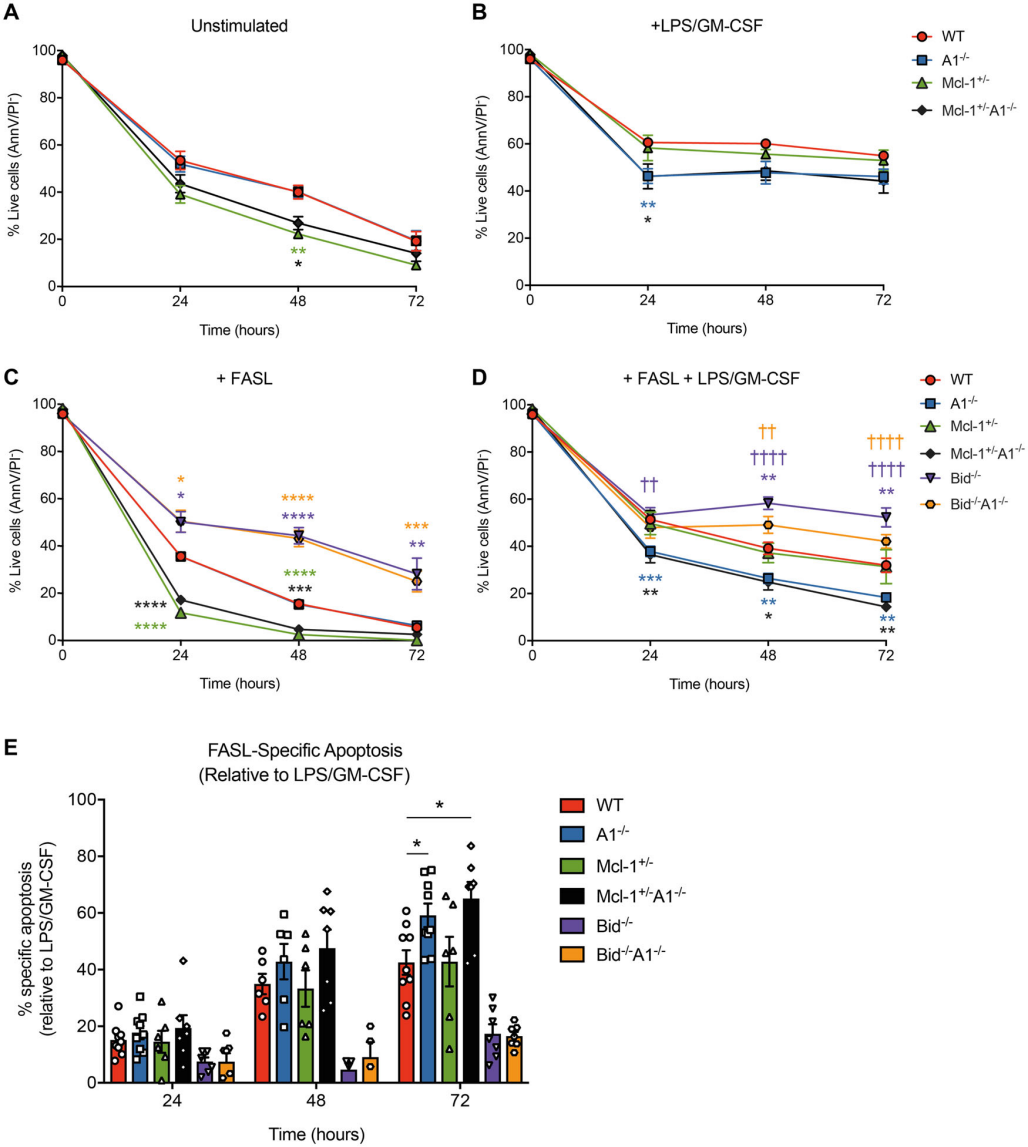
Survival analysis of neutrophils from mice with the indicated genotypes cultured in **a** simple medium (no added cytokines), **b** after stimulation with 10 ng/mL GM-CSF plus 10 ng/mL LPS, **c** after treatment with Fc-FASL (0.6 ng/mL), and **d** after stimulation with LPS plus GM-CSF (10 ng/mL each) and Fc-FASL (0.6 ng/mL). **e** FASL-specific apoptosis when compared to survival of cells stimulated with LPS plus GM-CSF. Data are from five combined experiments (WT *n* = 9, *A1*^*–/–*^ *n* = 9, *Mcl-1*^*+/–*^ *n* = 6, *Mcl-1*^*+/–*^*A1*^*–/–*^ *n* = 7, *Bid*^*–/–*^ *n* = 7, and *Bid*^*–/–*^*A1*^*–/–*^ *n* = 7 mice). Statistical significance (*P < 0.05, **P < 0.01, ***P < 0.001, ****P < 0.0001) was determined using Student’s *t*-test at each timepoint compared to WT (*) or *A1*^*–/–*^ (†).

Neutrophils are highly sensitive to FAS-induced apoptosis^1^, but this death is delayed when they are activated by LPS plus GM-CSF^1^. We analyzed FASL-induced apoptosis with and without LPS plus GM-CSF stimulation in neutrophils from *A1* and *Mcl-1* mutant mice. Additionally, FASL-induced apoptosis in neutrophils is dependent on caspase-8-mediated activation of the pro-apoptotic BCL-2 family member BID (called tBID)^10^, which A1 binds to with high affinity^11^. We therefore also included *Bid*^*–/–*^ mice^12^ as a control in our experiments and, furthermore, generated *Bid*^*–/–*^*A1*^*–/–*^ mice in order to examine whether any effects seen in the *A1*^*–/–*^ cells were dependent on A1–tBID interactions.

*Mcl-1*^*+/–*^ (and *Mcl-1*^*+/–*^*A1*^*–/–*^) neutrophils died quicker than wild-type cells after FASL treatment (Fig. 1c). FASL-induced apoptosis was greater than basal apoptosis in culture (Supplementary Fig. 3). *Bid*^*–/–*^ neutrophils were protected from FASL-induced apoptosis^10^. LPS plus GM-CSF protected both wild-type and *Mcl-1*^*+/–*^ neutrophils against FASL-induced killing (Fig. 1d). In contrast, *A1*^*–/–*^ and *Mcl-1*^*+/–*^*A1*^*–/–*^ neutrophils exhibited significantly more apoptosis across all time points after treatment with FASL in LPS plus GM-CSF-activated neutrophils. Taking into account the increase in apoptosis after LPS plus GM-CSF stimulation in *A1*^*–/–*^ neutrophils. We observed a trend towards more FASL-specific apoptosis in the A1-deficient cells, although this only reached statistical significance at 72 h (Fig. 1e). The amount of FASL-specific apoptosis did not differ between *Bid*^*–/–*^ and *Bid*^*–/–*^ *A1*^*–/–*^ cells, indicating that the increased sensitivity of activated *A1*^*–/–*^ neutrophils to FASL killing is mediated by tBID. *Bid*^*–/–*^*A1*^*–/–*^ neutrophils displayed lower viability than their *Bid*^*–/–*^ counterparts, both after LPS plus GM-CSF stimulation (Supplementary Fig. 4) and with the combination of LPS, GM-CSF, and FASL (Fig. 1d), fitting with the role we showed for A1 in promoting cell survival after LPS plus GM-CSF stimulation alone.

Collectively, we demonstrate that upregulation of A1 after stimulation imparts a survival advantage in neutrophils, including FASL-induced apoptosis. However, A1’s role is relatively small, and other factors must also regulate the survival of activated neutrophils. These results suggest a previously unrecognized role for A1 in promoting neutrophil survival in an inflammatory context.

## Supplementary information

Supplemental Materials
