# Supplementary material for: The pro-survival Bcl-2 family member A1 delays spontaneous and FAS ligand-induced apoptosis of activated neutrophils
Source: Cell Death Dis. 2020 Jun 18;11(6):474. doi: 10.1038/s41419-020-2676-9 (PMC7303176; doi:10.1038/s41419-020-2676-9)
Supplement: Supplementary file 1 — Supplemental Materials [file 41419_2020_2676_MOESM1_ESM.docx]

**Supplementary Information**


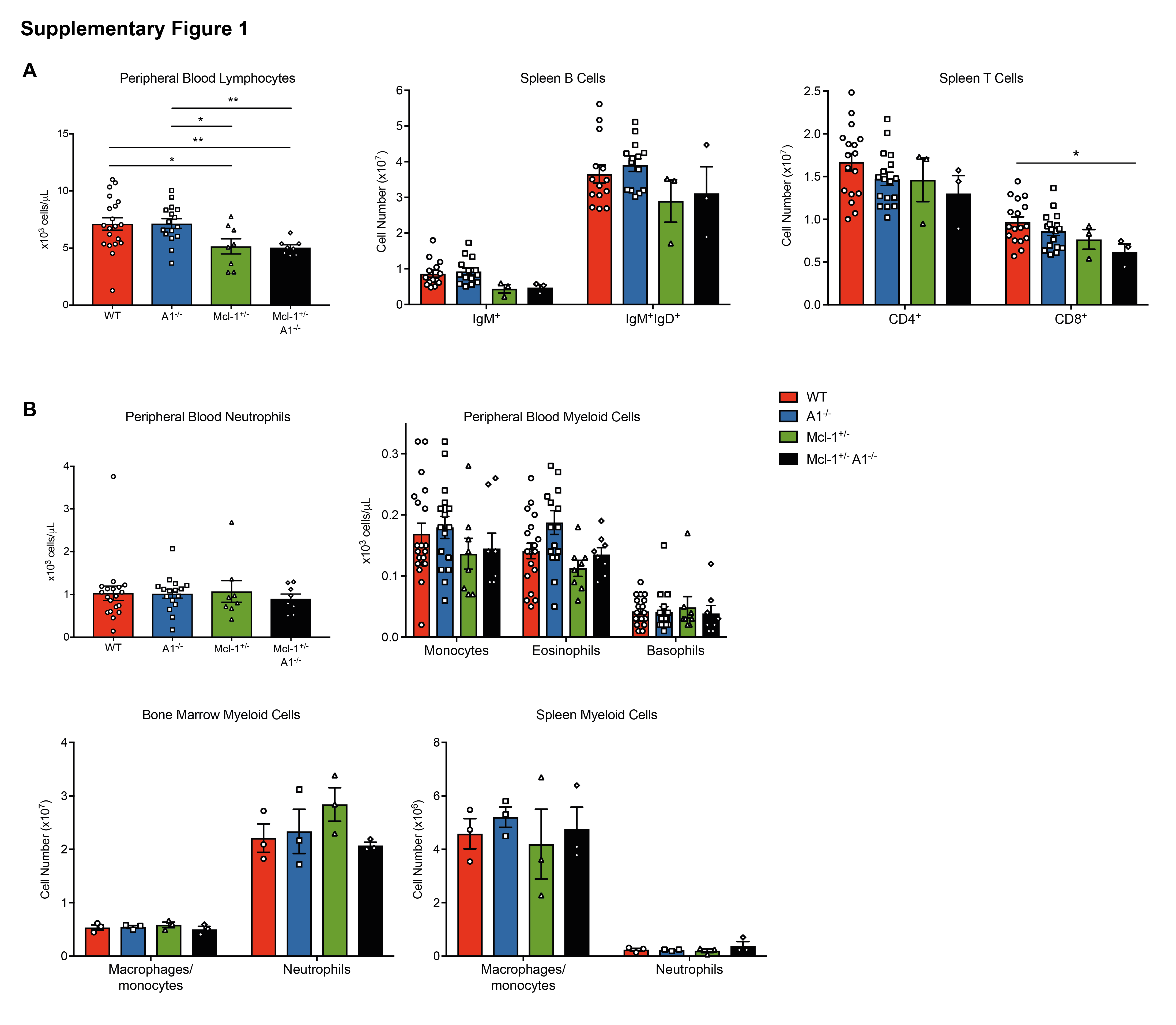


**Supplementary Figure 1: Haematopoietic cell analysis of *Mcl-1^+/­–^A1^–/–^*** **mice shows no differences compared to *Mcl‑1^+/–^* mice.**

Total numbers of lymphocyte **(A)** and myeloid cell **(B)** populations in the peripheral blood, bone marrow and spleens of WT, *A1^–/–^*, *Mcl-1^+/–^* and *Mcl-1^+/­–^A1^–/–^* mice. Peripheral blood cell numbers determined by ADVIA Blood Analyzer analysis; spleen and bone marrow cell populations determined using flow cytometry with the following cell surface markers: B220^+^IgM^+^IgD^-^ and B220^+^IgM^+^IgD^+^ B cell subsets, TCRβ^+^CD4^+^ and TCRβ^+^CD8^+^ T cell subsets, CD11b^+^ macrophages/monocytes and CD11b^+^GR-1^+^ neutrophils. Statistical significance (P < 0.05) determined using student’s t-test. Each dot represents one mouse.


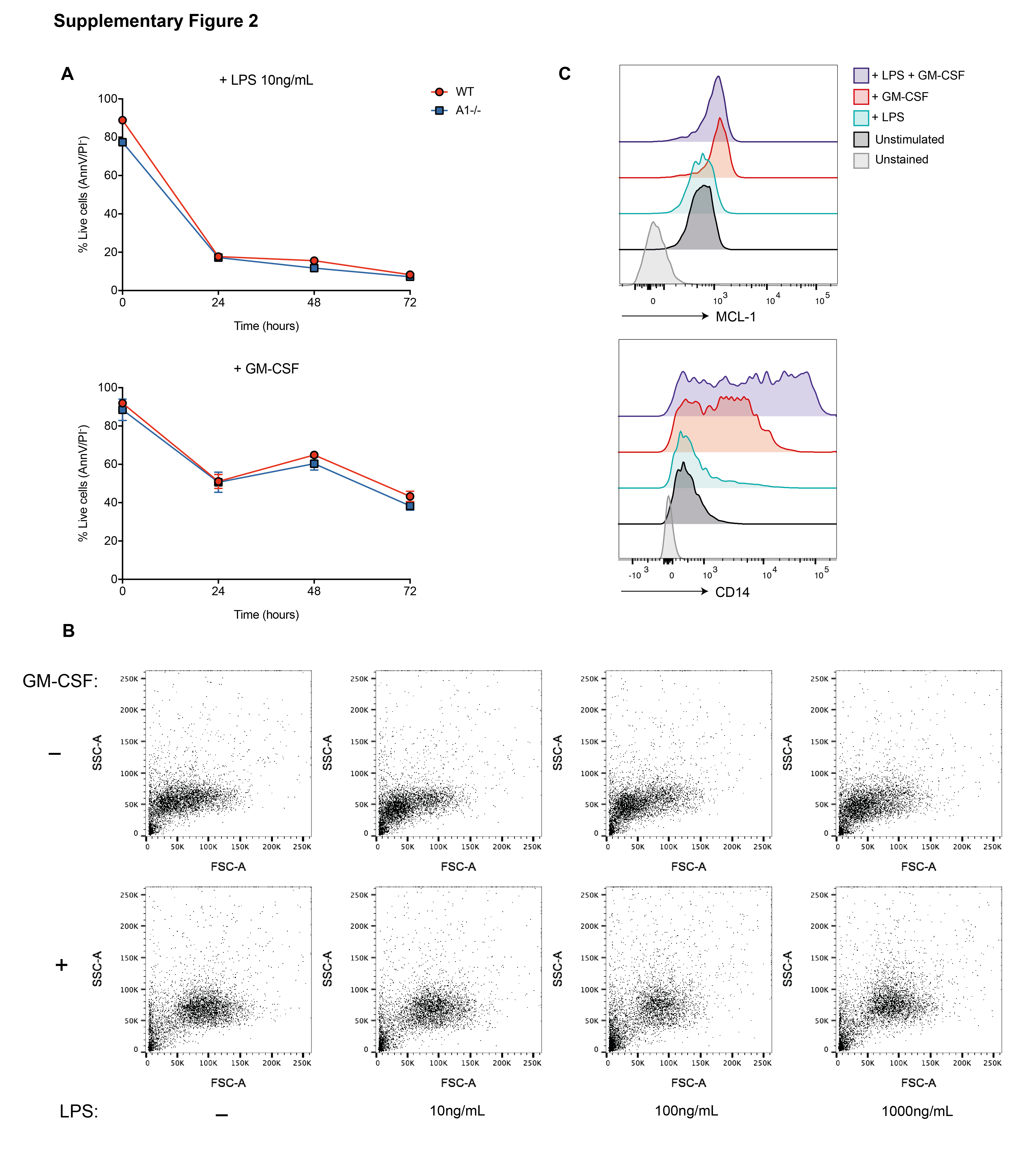


**Supplementary Figure 2: LPS stimulation alone does not activate neutrophils *in vitro*.**

**(A)** Survival analysis of WT and *A1­^–/–^* neutrophils in culture after stimulation with 10 ng/mL LPS alone (top panel) or 10 ng/mL GM-CSF alone (bottom panel). LPS treatment representative of one independent experiment, with n=1 mouse per genotype. GM-CSF represents 3 independent experiments combined, with n=7 WT and n=6 *A1­^–/–^*. **(B)** Forward- and side-scatter plots collected by flow cytometry of WT neutrophils stimulated with LPS (0 – 1000 ng/mL) with or without the addition of GM-CSF (10 ng/mL). Data collected from the same experiment as (A). **(C)** Intracellular FACS analysis of MCL-1 expression and CD14 levels on the surface of WT neutrophils stimulated with LPS alone (10 ng/mL), GM-CSF alone (10 ng/mL) or the combination of LPS plus GM-CSF. Data collected in the same experiment as (A), n=1.


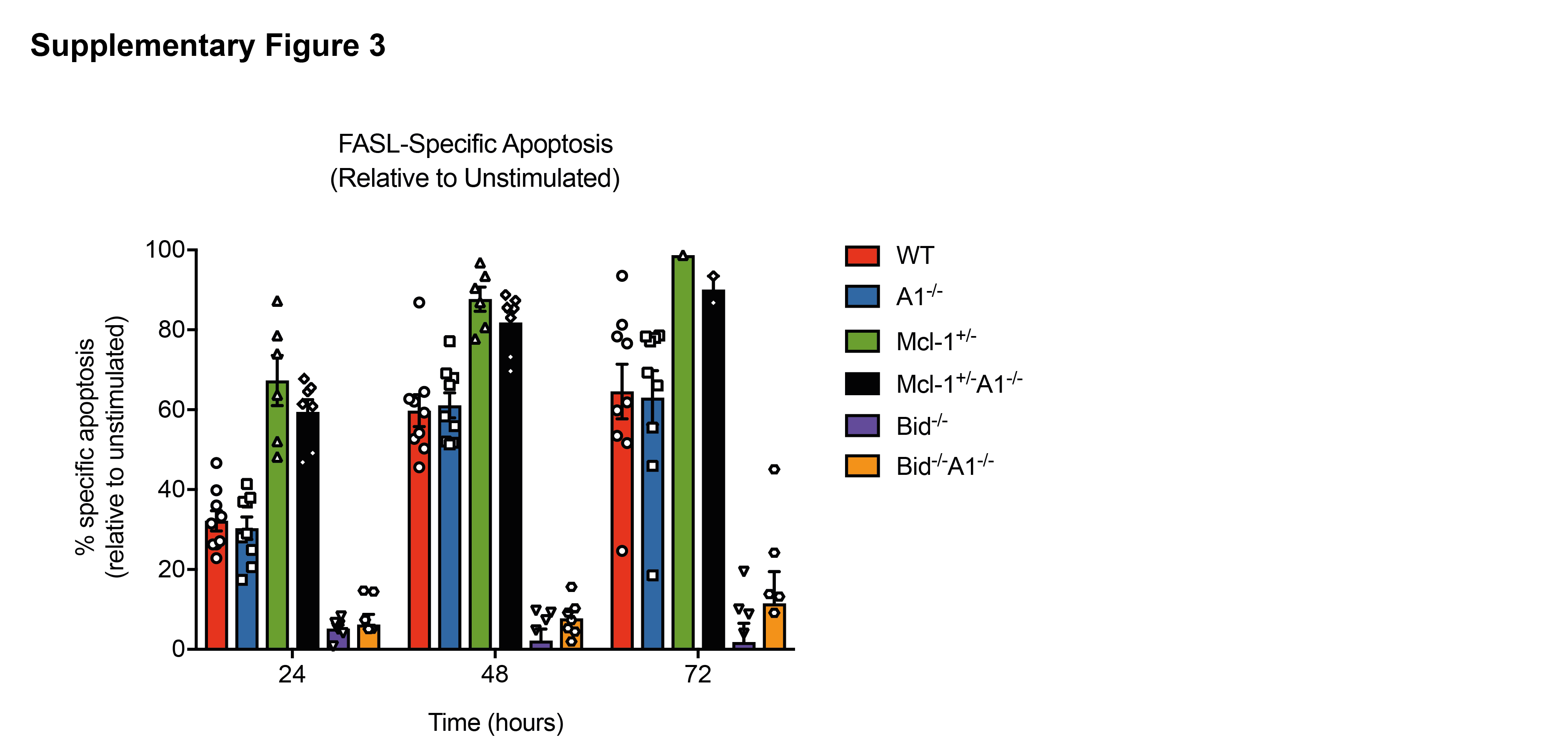
**Supplementary Figure 3: *Mcl-1^+/­–^* neutrophils undergo more FASL-specific apoptosis and *Bid^–/–^* neutrophils are protected from FASL-specific apoptosis.**

FASL-specific apoptosis across the measured timepoints in neutrophils of the indicated genotypes, calculated by the amount of apoptosis in unstimulated cells compared to FASL‑treated cells.

**
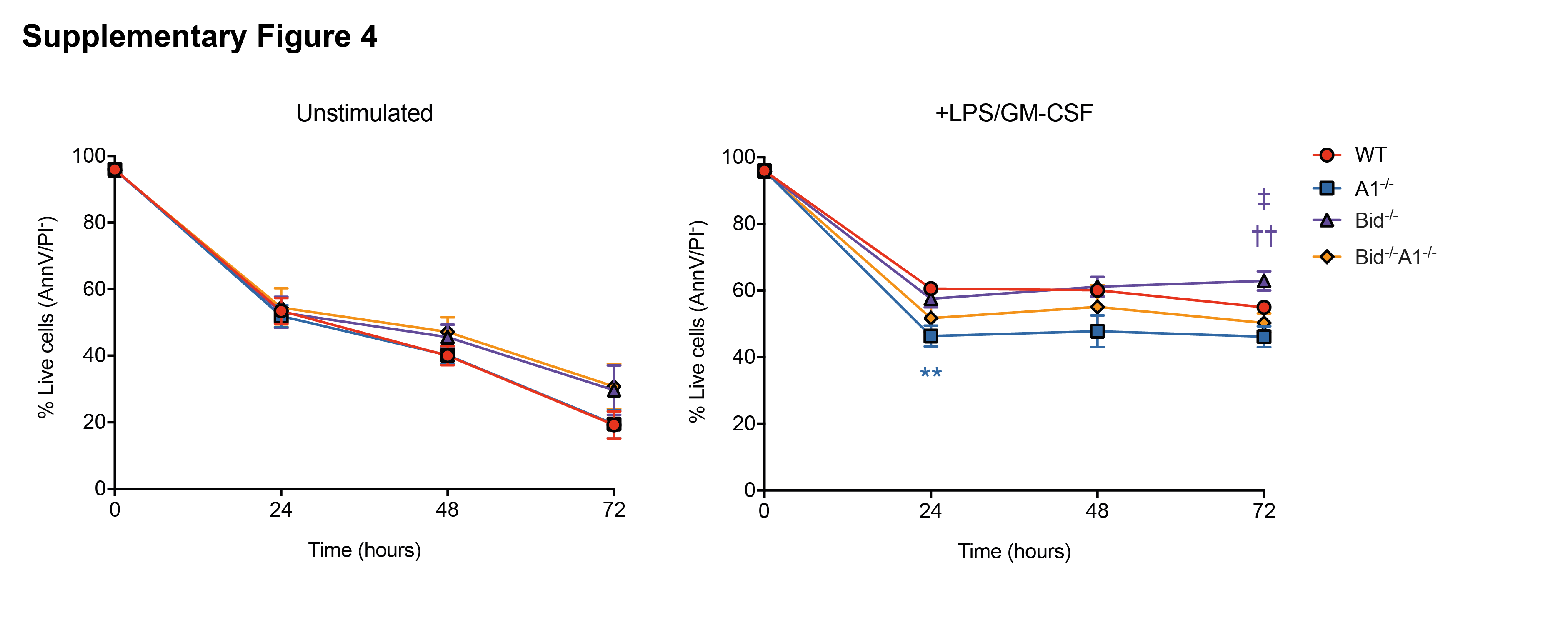
Supplementary Figure 4: Survival of *Bid^–/–^* and *Bid^–/–^ A1^–/–^* neutrophils in culture and after stimulation with LPS plus GM-CSF.**

Survival analysis of neutrophils isolated from mice with the indicated genotypes cultured in simple medium (no added cytokines, left panel) or after stimulation in culture with 10 ng/mL GM-CSF plus 10 ng/mL LPS (right panel). Data are from 5 combined experiments (WT n=9, *A1^–/–^* n=9, *Mcl-1^+/–^* n=6, *Mcl-1^+/­–^A1^–/–^* n=7, *Bid^–/–^* n=7 and *Bid^–/–^A1^–/–^* n=7 mice). Statistical significance (* = P < 0.05, ** = P < 0.01) was determined using student’s t-test at each timepoint compared to WT (*), *A1^–/–^* (†), or *Bid^-/-^* (‡).

**Methods**

1. Mouse models

All animal experiments were approved by The Walter and Eliza Hall Institute of Medical Research Animal Ethics Committee and were conducted in accordance with the Prevention of Cruelty to Animals Act (1986) and the Australian National Health and Medical Research Council Code of Practice for the Care and Use of Animals for Scientific Purposes (1997). *Mcl‑1^+/–^A1^–/–^* and *Bid^–/–^A1^–/–^* mice were generated by intercrossing *Mcl-1*^+/–^ or *Bid^–/–^* mice, respectively, with *A1*^–/–^ mice^3^. All mouse strains have been generated on and were maintained on a C57BL/6 background.

1. Haematopoietic cell analysis and flow cytometry

Peripheral blood was analysed with the ADVIA automated haematology system (Bayer). Spleen and bone marrow were harvested from mice (8-12 weeks old) and cell populations identified using flow cytometry. The following fluorochrome-conjugated monoclonal antibodies (produced in-house, BD Biosciences or BioLegend) were used: IgM (5.1), IgD (11‑26C), B220 (RA3-6B2), TCRβ (H57-597), CD4 (GK1.5), CD8 (53-6.7), CD11b/MAC-1 (M1/70), GR-1 (RB6-8C5). Flow cytometry data were analysed using FlowJo (Treestar). Cell numbers were calculated from total organ cell counts, collected with the CASY Cell Counting system (OMNI Life Science). Intracellular flow cytometry was performed using cells fixed and permeabilized using the FOXP3 fix/perm kit (eBiosciences). MCL-1 (clone 19C4-15^4^) and CD14 (Sa14-2) antibodies were used.

1. Neutrophil enrichment

Neutrophils were isolated from bone marrow of mice (>8 weeks old) with Stem Cell Technologies’ Mouse Neutrophil Enrichment Kit, according to the manufacturer’s instructions. Neutrophil purity (CD11b^+^GR-1^+^) was routinely confirmed by flow cytometry at >90%.

1. Survival assays

Neutrophils were seeded at 3x10^4^ cells/well in 96-well plates in RPMI medium (plus 10% FCS Sigma-Aldrich (v/v), 50 µM β-mercaptoethanol, and 100 U/mL penicillin/streptomycin) with or without 10 ng/mL GM-CSF (recombinant mouse GM-CSF produced in-house^5^). Cells were incubated for 1 h at 37°C with 10% CO_2_ before adding medium alone or medium containing LPS (10 ng/mL; ultrapure LPS from E. coli 0111:B4 strain, InvivoGen), recombinant Fc-FASL (0.6 ng/mL, produced in-house as described^6^), or LPS plus GM-CSF plus Fc-FASL and incubated for the specified times. Treatments were performed in triplicate.

Live cells were identified as Annexin-V^–^/PI^–^ by flow cytometry. Cells were washed and resuspended in Annexin-V binding buffer (2.5 mM CaCl_2_, 0.14 M NaCl and 0.01 M HEPES pH 7.4) containing Annexin-V conjugated to FITC (Biolegend) and propidium iodide (PI, 5 µg/mL). FASL-specific apoptosis was determined as follows:

% apoptotic cells = 100% – %(Annexin-V­^–^ PI^–^)

% “spontaneous” apoptosis = % apoptotic cells in unstimulated controls, or LPS/GM-CSF treatment (when determining FASL-specific apoptosis in background of LPS/GM-CSF stimulation)

$$\text{\% FASL-specific apoptosis = 100}\text{\%}\text{ ×}\frac{\% \text{FASL-induced apoptosis}-\% "\text{spontaneous}"\text{ apoptosis}}{100-\% \text{"spontaneous" apoptosis}}$$

1. Statistical Analysis

Statistical significance was identified using student’s t-test (Prism Software, Graphpad). Data are represented as means ± SEM.

**References for Supplementary Material:**

1. Vikstrom, I. *et al.* Mcl-1 Is Essential for Germinal Center Formation and B Cell Memory. *Science* **330**, 1095–1099 (2010).

2. Kaufmann, T. *et al.* The BH3-Only Protein Bid Is Dispensable for DNA Damage- and Replicative Stress-Induced Apoptosis or Cell-Cycle Arrest. *Cell* **129**, 423–433 (2007).

3. Schenk, R. L. *et al.* Characterisation of mice lacking all functional isoforms of the pro-survival BCL-2 family member A1 reveals minor defects in the haematopoietic compartment. *Cell Death Differ.* **24**, 534–545 (2017).

4. Okamoto, T. *et al.* Enhanced stability of Mcl1, a prosurvival Bcl2 relative, blunts stress-induced apoptosis, causes male sterility, and promotes tumorigenesis. *Proc. Natl. Acad. Sci. U. S. A.* **111**, 261–266 (2014).

5. Metcalf, D. *et al.* In vitro actions on hemopoietic cells of recombinant murine GM-CSF purified after production in Escherichia coli: comparison with purified native GM-CSF. *J. Cell. Physiol.* **128**, 4^3^21–431 (1986).

6. O’Donnell, J. A. *et al.* Fas regulates neutrophil lifespan during viral and bacterial infection. *J. Leukoc. Biol.* **97**, 321–326 (2015).
